# Physical Therapy Intervention for Individuals with Rett Syndrome

**DOI:** 10.1100/tsw.2006.187

**Published:** 2006-10-10

**Authors:** Meir Lotan, Susan Hanks

**Affiliations:** ^1^ National Evaluation Team, Israeli Rett Center , Chaim Sheba Medical Center, Tel HaShomer, Ramat Gan, Israel; ^2^ Zvi Quittman Residential Centers, Elwyn, Jerusalem, Israel; ^3^ Department of Physical Therapy, Academic College of Judea and Samaria, Ariel, Israel; ^4^ Child Development and Rehabilitation Center, Oregon Health and Science University, Portland, OR, USA

**Keywords:** Rett syndrome, physical therapy, intervention, neuromuscular limitations

## Abstract

Individuals with Rett syndrome (RS) present a vast array of orthopedic and neurological difficulties. Typical problems, which may need to be addressed, when treating this population are functional limitations, low cardiovascular capacity, hypotonia, ataxia, apraxia, loss of transitional movements, spasticity, scoliosis and/or kyphosis, loss of ambulation, loss of hand function, foot deformities, and spatial disorientation. Coping with such difficulties and overcoming the associated limitations carry a wearisome task for the individual with Rett as well as for her family. An informed and intensely applied physical therapy regime can help the child and the family cope and even overcome the above-mentioned limitations. The present article presents some insights regarding the intervention with individuals with RS, an overview of typical neuromuscular problems associated with RS, and appropriate suggestions pertaining to clinical intervention that have been found to contribute to this populations well-being. The information presented is mainly based on the clinical knowledge of the authors.

## INTRODUCTION

Rett syndrome (RS) is a genetic disorder that primarily affects females[1,2]. The disorder causes a neurological and developmental arrest that manifests itself in a variety of disabilities, such as loss of functional hand use, loss of acquired speech, apraxia, ataxia, autonomic system dysfunction, epilepsy, breathing abnormalities, failure to thrive, and muscle tone irregularities[3,4,5]. In order for the children and women with RS, and their families, to live life to the fullest, proper intervention should be applied. The present article addresses the physical difficulties typically exhibited by individuals with RS and suggests possible intervention techniques, according to the scarcely existing literature and based mainly on the experience of the authors.

## PHYSICAL THERAPY FOR RETT SYNDROME

Due to the longevity of individuals with RS[6] and due to the complex nature of the RS disability, physical therapy is an important part of the management of the disorder. Individuals with RS show a considerable functional diversity. Some girls might never achieve independent sitting or standing, while a minority of children may gain high functional abilities such as running, skiing, and trampoline jumping[7]. Due to such versatility, a thorough evaluation should be performed with each child entering an intervention program. After such an evaluation, preferably by a multidisciplinary team[8,9], an intervention program should be drawn up, specifically tailored for each client. In many cases, the child with RS is treated by a team of therapists from fields of expertise such as physical therapy, occupational therapy, speech therapy, music therapy, hydrotherapy, and hippotherapy (horseback riding). Each of the disciplines involved in the therapeutic program uses a combination of different techniques intended to maintain and maximize function of the individual with RS. While these therapies do not cure RS, they can help the client by lessening the difficulties experienced by her, thus helping the individual with RS and her family to cope with the above-mentioned limitations[10].

Nevertheless, it is vital for the success of the intervention program that different members of the team coordinate their therapeutic efforts into a combined management approach in accordance with all team members' agreement (including the parents and the child with RS). A well-planned physical intervention is of great importance to individuals with RS[11,12]. Such a program may maintain or improve function, prevent deformities, and provide positioning and mobility[12], thereby contributing to the social accessibility of the client. It is important to remember that while individuals with RS share many similarities, their problems and response to intervention may vary dramatically. The basic goals of physical therapy are to:

- Maintain or increase motor skills
- Develop or maintain transitional skills
- Prevent or reduce deformities
- Alleviate discomfort and irritability
- Improve independence

The physical therapist may assess and then improve walking and sitting patterns, monitor changes over time, and advise the educational team and parents on topics such as adapted seating systems, assistive technology, and mobility devices.

Regarding the physical therapy program, however, the therapist is in dilemma. On one hand, it is known that functional and clinical advancements of individuals with RS have been reported several times by different intervention teams, during childhood as well as in adulthood[12,13,14,15,16,17], yet on the other hand, it is important to understand that each individual with RS has limits of achievement that are determined by her underlying neurological situation. Therefore, the physical therapist should advise parents regarding that which is reasonable to expect from a child or adult with RS. As much as all professionals want their clients to achieve a high level of function, it is also important to respect what is possible and not spend time and resources on goals that cannot be met at the expense of other aspects of school and family life. This type of understanding may require time and input from the parents, pediatrician, orthopedist or neurologist, and therapist.

Due to the fluctuating nature of RS[12,18] and to external influencing factors (such as surgery or fracture), the child might be occasionally exposed to periods of inactivity. Enhanced physical therapy intervention is warranted after illness, fractures, or surgery, or whenever special effort is made to regain skills that the individual previously possessed[19].

Despite the above-mentioned variability in physical abilities among individuals with RS, there are some neuromotor problems typical for many with this disorder. Such common occurrences should be taken into account when treating the individual with RS. This article will present such common problems as well as suggest appropriate intervention approaches.

## BASIC STEPS WHEN APPROACHING A CHILD WITH RS

When considering intervention for an individual diagnosed with RS, therapists must have an understanding of the complex problems associated with the four stages of the condition in order to develop appropriate management plans. A variety of treatment strategies can be applied at a given stage and may be helpful in achieving or maintaining a level of independence in a particular functional area. The therapist must assume a key role in instructing parents and teachers in the handling of a child with RS.

Andreas Rett (1924–1997) himself[20] reminded the professionals of the responsibility to care for, nurture, and advance these special children. For the therapist involved with the child and family, such responsibility carries with it a commitment to develop practical solutions to daily problems in caring for the child with RS. This approach, as much as it is true for most children with disabilities, is greatly emphasized for individuals with RS. The daily and periodic fluctuations in mood and function, and the constantly evolving nature of this syndrome (development of scoliosis, loss of mobility, and eruption of epileptic seizures) can sometimes discourage even the experienced physical therapist. Thus, the intervention plan should be flexible and constantly adjusted to the child’s state. Furthermore, the therapist should always be sensitive to the needs of the family and give appropriate advice and support when needed.

Individuals with RS usually show age-appropriate emotional abilities[21,22] and those abilities should be addressed by the therapist while treating the child and even before intervention initiation[23]. Before the beginning of any intervention, the physical therapist should familiarize him/herself with the child and a short opening ritual is recommended at the beginning of each therapeutic session[22]. During therapy sessions, it is advisable to allow the child to gain control over the sessions by enabling her to choose her preferred activity via an appropriate communication device (see Fig. 1).

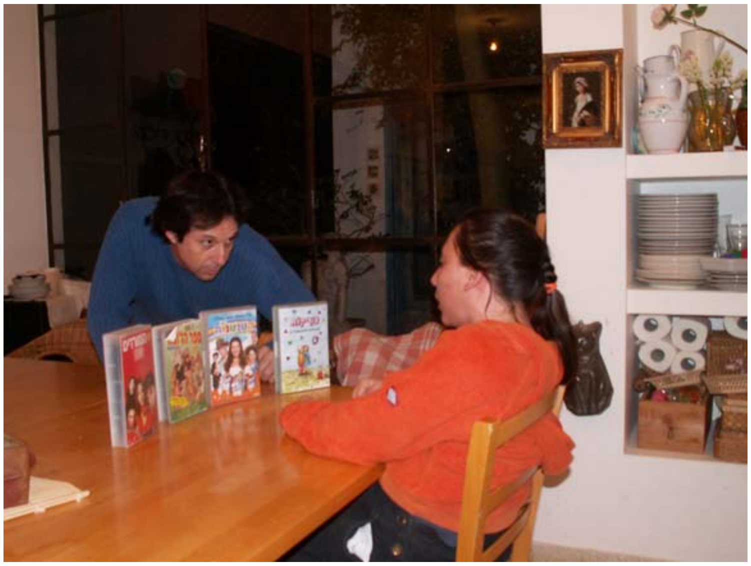

**FIGURE 1.** Empowering the child by enabling choice before initiation of physical therapy sessions.

The intensity of activities should be adjusted according to the reaction of the child. Some girls with RS show fear of movement and strongly resist external facilitation. If the child is having a difficult time adjusting to physical activity, the pace of the intervention should be adjusted and motivational factors, such as music, switch-operated games, video tapes, pets[24], and/or family members, should be included in the session.

## GUIDELINES FOR PHYSICAL INTERVENTION

Over many years of working with females with Rett syndrome, we have developed the following guidelines:

- The individual with RS is essentially controlled by her body instead of controlling it. Therefore, in most cases, physical activity by an external facilitator will not be extremely enjoyed by individuals with RS. The therapist should approach the child by initiating interpersonal bonding. Try and pace yourself with the child, conveying serenity and security. It is preferred that you commence physical exercise only after establishing basic trust.
- In physical intervention necessitating manipulation, apply caution since individuals with RS have been reported to have low bone mineral density (i.e., "fragile bones")[25,26,27] and a delayed reaction to pain, due to high pain threshold[10,28] or long reaction time to pain[29]. For this reason, the therapist might not be aware if the child is in pain.
- The major functional setback of the client with RS is apraxia, therefore verbal guidance should precede and accompany the performance of a specific task. The therapist's hands should gently, but decisively, direct the child's body to the requested goal and do all this without altering the client's personal pace. Trying to rush the individual with RS will most likely lead to her stagnation.
- 87% of individuals with RS display difficulties in coping with external facilitation[30]. This fear of movement must be addressed by the therapist and the approach must be holistic despite his/her engagement in physical intervention. The discomfort of the client and the tension her body presents should be eased before active participation is demanded.
- Music, especially familiar songs, is an excellent motivational factor[31]. Using live or recorded songs can relax the client with RS, easing the intervention for child and therapist alike[22].
- The progression of RS has a tendency towards dynamic fluctuations[18]. During one week, the child may walk and constantly advance in her ambulation abilities and by the next week, she might refuse to even stand on her feet. The attentive physical therapist should be flexible and flow with the unexplained changes, updating the goals to address new issues, while constantly evaluating the child's state and abilities as well as trying to maximize them.
- Physical intervention was found to improve the concentration of individuals with RS[18]. Therefore, it is recommended to plan educational tasks closely following physical interventions (hydrotherapy, physical therapy, occupational therapy).
- Enhancing the child's desire to move and explore her surroundings can be achieved by stimulating her in various ways, such as:
  - Talking to her (most individuals with RS are extremely fond of soft whispering)
  - Diverting her attention to peers and events in her vicinity
  - Surrounding the person with interesting objects (such as those that shine or flicker in a dark room – Snoezelen, big mirrors on the walls)
  - Giving ample opportunities for tactile experiences
  - Using interesting gadgets (like voice- or hand-operated communication devices and household appliances, computer programs)
  - Supplying switch-operated toys
  - Suggesting water games
- Children with RS who have learned how to walk at advanced ages have been described in the past on several occasions by parents. Few studied have reported several individuals with RS to regain walking[13,14]. In some cases, adult women with RS have regained walking abilities after 15 or 20 years of wheelchair usage[12,15]. Some children with RS exhibit a marked distortion of their orientation in space and have been reported to regain walking abilities in several instances due to intensive "overcorrection" intervention[13,32]. In regards to the previous data, the connection between walking ability and severity of scoliosis has been numerously mentioned by different authors[3,33,34]. Furthermore, the disadvantages of a sedentary lifestyle for all populations have been constantly mentioned in medical literature[16,35]; therefore, individuals with RS should always be directed towards walking.

- When walking with the individual with RS, always give the minimal amount of support required for the safety of the client, since in most cases she will try to lean on the person holding her in order to ease her efforts[36].
- Losing the ability to walk can originate from the development of scoliosis, deformed feet[3,12], general medical deterioration, tone changes, muscle shortening, and the loss of midline orientation[13]. Due to secondary dangers of long-lasting immobility, such as loss of function, reduction in muscle strength, reduction in body and limb range of motion, the development of contractures, and reduction in cardiopulmonary fitness[37,38,39], the therapist should be alert to changes affecting ambulation. When such changes are observed, interventional goals should change accordingly and enhanced efforts should be invested in walking programs along the day.
- When the child is nonambulatory, a daily program in a standing frame is mandatory[34]. Such a program can assist in the maturation of the hip joints, thereby reducing the chance for hip dislocation, reducing osteoporosis and other associated problems[40,41,42], increasing the child's postural control, as well as introducing the child to healthier alternatives to sitting[4]. Standing should always challenge the child's postural abilities; therefore, the use of the most active systems should be warranted (see Figs. 2 and 3).
- Due to apraxia, the functional abilities of individuals with RS improve with continuous practice. The emphasis of practice should be placed on chores that the individual with RS finds rewarding (putting rings in a box has no purpose, but taking cookies out of a jar does, at least for the child, and is an action that demands planning and the execution of a motor act, fine motor abilities, and hand-eye coordination).
- Apraxia may lead to a situation where an individual with RS is able to perform a functional task, such as getting up from the floor to a standing position in a very specific manner and even only in one familiar place[12]. It is therefore important to identify such conditions and to use them as a fulcrum for further generalization and development.
- Some individuals with RS demonstrate agitation when confined to small or crowded rooms with no opportunity for free movement. Such situations are better avoided.
- Due to the difficulty of some individuals with RS to adjust to changes in routine[23], it is advised to keep the structure of the intervention stable. When changes are due, inform the child in advance and gradually implement the planned adjustments.
- In many developmental diseases, intervention is gradually reduced as the child gets older. As individuals with RS grow older, their body stiffens and muscle spasms increase, their chance to develop scoliosis becomes essentially higher. In such circumstances as well as taking into account the longevity of individuals with RS, it would be logical to preserve or increase intervention intensity[19].
- Intervention for individuals with RS should be implemented through a complete and comprehensive program accompanying the child throughout her daily routine — a total program[12]. Such a program casts a therapeutic net around the child and constantly combats the difficulties caused by RS (i.e., a total program means that the child is properly seated while at home, at school, and on the way to and from these places; every caregiver will always demand of the child that she perform at her highest capacity; feeding habits of the child are known and similarly addressed by the parents, grandparents, siblings, school facilitators, and anyone who might contribute to the child's care). The authors would like to assert that any such program, however helpful for the child, can only be performed within the capacity of the human environment taking care of the individual with RS; therefore, the physical therapist should be aware of such limitations when aiming to establish such a program, keeping it within realistic boundaries.

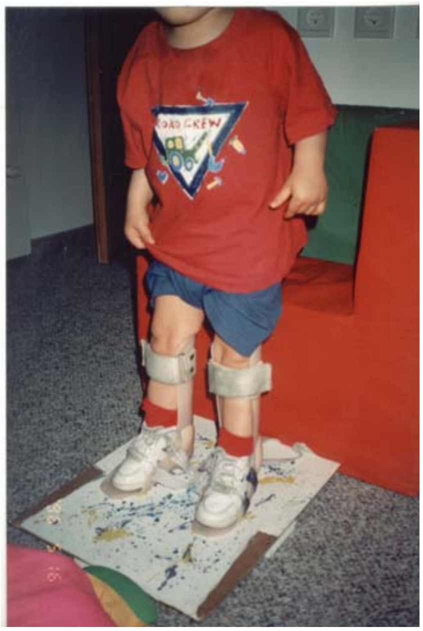

**FIGURE 2.** A "splint standing frame".

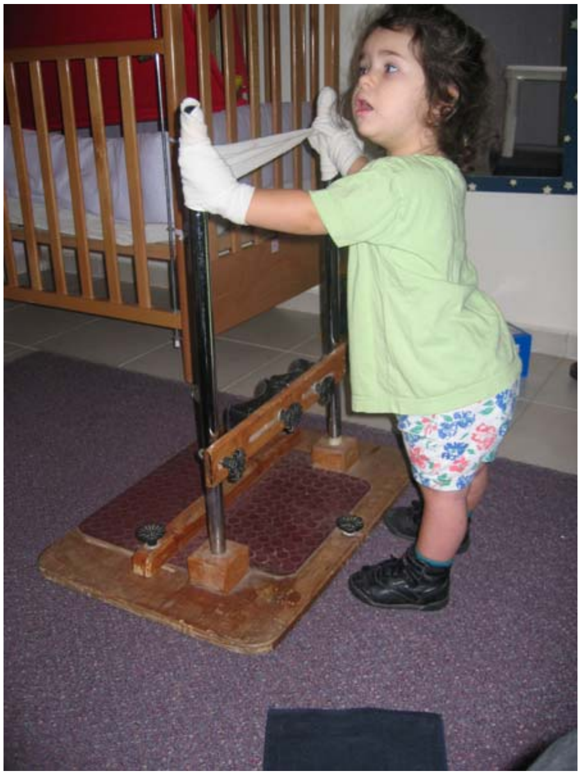

**FIGURE 3.** An active standing frame.

## SUGGESTED PHYSICAL THERAPY GOALS

The goals mentioned below should be specified for each child after a thorough individual evaluation, during which the therapist should carefully evaluate the person's abilities in order to detect obstacles impairing her function. These obstacles can be modified through facilitation of movement, tone reduction activities, and balancing responses. It is reiterated that an intensive therapeutic program should envelop the client's daily activities and surroundings, as well as to set clear intervention tasks for anyone taking care of the child. Regardless of the importance of such a program, the client's tolerance must be considered at all times, expecting that she will probably resist being moved or manipulated[43].

In spite of the phenotypic variability of individuals with RS, our experience suggests that the following physical therapy goals may be appropriate for many of them:

- Normalize muscle tone
- Advance articular range of motion
- Reduce apraxia by repetitive functional experiences
- Increase and improve cardiovascular fitness by applying an appropriate program
- Stimulate hand use by challenging the child to climb, swing, and/or support herself
- Achieve better balance reactions through exercises and daily activities
- Promote better coordination and balance through practice in various situations and environments
- Reduce setbacks cast by ataxia by training the child through different, gradually increasing postural challenges
- Improve body awareness by deep proprioceptive input and active motion
- Reverse progression of scoliosis through proper intervention (see below)
- Maintain and improve mobility
- Increase protective responses

## INTERVENTION ACCORDING TO RETT SYNDROME STAGE

### Stage I

Onset — Most children at this stage are not yet diagnosed as having RS. Most often, they are diagnosed as having hypotonic cerebral palsy, autism, or both[44]. The child will display characteristics such as delayed developmental milestones; reduced hand function, play skills, and communication; the child may appear easy to handle and quiet. Since physical therapy intervention is symptomatic, the therapeutic program will address the problems the child presents. Usually, the response to stage I typical stagnation is to enroll the child in a therapy program. In many cases, some improvement will be noted during this stage, although the ultimate gradual deterioration is undeniable. Specifically, it is realistic to work toward independent standing and even ambulation, since many of the girls appear to maintain ambulation attained in stage I over a long period of time. On the other hand, the outcome of therapy aimed at developing other functional skills may be found less positive, yet should not be avoided.

### Stage II

The rapid destructive phase — The loss of acquired developmental milestones is the significant characteristic of stage II. This rapid deterioration may pose a difficult management problem for the family and the specialist team involved. The child may still not be diagnosed as having RS or a final diagnosis may still be pending. The child who is involved in a therapy or educational program is not making progress or is losing ground. The stereotypical hand movements are fully present at this time. Additional medical problems may become apparent; these can include seizures, deceleration of head growth, irregular breathing or hyperventilation, and initiation of spinal asymmetry. In order to alleviate the chaotic nature of this short stage[18], relaxing techniques in different environments have been suggested[23,45]. Gentle handling of the child at this stage has been recommended as a means of reducing sensory intake; this approach was hypothesized to benefit brain development[46,47]. Such an approach can also enhance bonding between the therapist and child, and will yield positive results in future therapeutic interventions.

## Stage III

Plateau — Stage III is a lengthy stage in RS, characterized by a relatively calm period wherein intervention aimed at advancement is warranted. Nevertheless, during this stage, most of the individuals with RS develop significant problems, such as deformities and contractures (which require shoe inserts, orthoses, and a daily intervention program; see below for more information)[12]. If scoliosis is present, it can progress rapidly and might require surgery in 25% of mobile females with RS as well as in 61% of nonmobile females[33]. Stereotypical hand mannerisms are obvious and usually accompanied by minimal hand function. The use of splinting and intensity of splint usage is controversial among different practitioners. Some will generally refrain from splinting the child’s hands as a regular intervention, while others believe that splinting often improves the child’s function and enhances her awareness to the environment. Nevertheless, the authors agree that splinting or arm restraints have been found helpful in the following cases:

- Situations where skin problems develop as a result of constant hand-to-mouth behavior.
- When restraining the less-active hand, it was found that the individual with RS increases control over her other hand.
- When the stereotypical movements prevent the child from being properly fed (i.e., if the child is constantly putting her hand in her mouth throughout meals).
- When it disrupts the child’s social interactions. (For example, a child with RS was integrated into a regular school, but was not approached by normal peers due to constant drooling and spitting over her hands. The use of splints, preventing her from putting her hand in her mouth, turned things around and the child was immediately accepted by her peers.)

## Stage IV

Late motor deterioration — Significant decrease and lack of mobility is characteristic of stage IV. Many of the individuals with RS, although nonambulatory, may still be able to participate in a supported transfer (e.g., moving from a wheelchair to a bed). Individuals that constantly use a wheelchair will typically present a worse phenotypic expression, consequentially requiring more care. Individuals with RS at this stage are often described as having improved emotional contact and better-controlled epilepsy. In some cases, however, additional medical issues are encountered. Such problems may include worsening scoliosis and contractures, lower extremities atrophic changes[4], and circulatory (cold extremities) and feeding problems (e.g., independent eating, reflux, constipation)[48,49]. Growth retardation will usually be more apparent since most individuals with RS are of short stature and are slim[50]. At this point, the physical therapist should provide extended therapeutic support and educate involved individuals (i.e., therapists, caregivers, available family members) in regards to a daily exercise routine aimed at improving mobility, range of motion, transfer skills, positioning, and lifting of the child. If it is within the professional expertise of the therapist, he/she should evaluate and customize a properly supported seating system. The physical therapist’s role should be favorably considered in pre- and postsurgical follow-up and management[12,19].

## SPECIFIC MOTOR PROBLEMS IN RETT SYNDROME

RS is a disorder that presents an array of neuromuscular limitations that need proper therapeutic intervention. Specific motor problems associated with RS, which may need to be addressed by the physical therapist, are changing (over time) and fluctuating muscle tone (hypotonia, spasticity, dystonia)[51], ataxia, motor apraxia, loss of transitional movements, scoliosis and/or kyphosis, loss of ambulation, reduced hand function, foot deformities, and spatial disorientation. In this section of the article, the development of most of these difficulties will be mentioned and intervention techniques and hints will be suggested.

### Muscle Tone

As with other characteristics of RS, variability among individuals is the norm. The infant with RS will typically show hypotonia or the muscle tone of the child with RS will be within the norm. With age, most individuals with RS change from being hypotonic to becoming hypertonic (spasticity usually starting from lower extremities). Of adults with RS, 30% remain hypotonic, 40% show spasticity as their main tonal characteristic, and 30% become dystonic[51]. These changes influence the handling of the child and therefore necessitate ongoing evaluation of muscle tone by the therapist. When tonal changes are observed, the physical therapist should alert other team members involved in caring for the child, while at the same time initiating the proper handling instructions for the educational team and for the parents.

When muscle tone of the individual with RS gradually changes from low to high, the change might cause a unique phenomenon; in contrast to children with cerebral palsy (toe walking), the child with RS presents an asymmetrical reaction, causing the child to side-tilt the trunk, causing initial development of asymmetry of the spine, eventually leading to scoliosis. Another unique reaction of individuals with RS to the shortening of the Achilles tendon could be the tilting of the pelvis backwards, causing the child to lose the ability to walk independently. Such events should be expected and dealt with in order to prevent their severe consequences. If spasticity is severe and becomes a constant disruption to function, other physical interventions such as hydrotherapy (with emphasize on slow movement in the water), serial casting, daily muscle stretching, and deep pressure stimulus could all be found helpful in the management of high muscle tone. Continuous intrathecal baclofen infusion was found to enhance functional benefits among various populations with high muscular spasticity[52], not including individuals with RS. This type of intervention may be considered in severe cases.

### Deformities

Because of the abnormal muscle coordination and spasticity typical of RS, some muscles are overactive and pull certain joints out of balance. This happens most commonly about the ankle and hip, especially when the child lacks the ability to ambulate[53]. Over the ankle, the excessive pulling of muscles (gastrocnemius and tibialis anterior) may cause severe plantar flexion with supination (see Fig. 4).

In some cases, where the adult with RS is ambulatory and shows fixed deformities that neither interfere with balance nor cause calluses, these deformities may be left alone. But since such deformity may interfere with the future standing and walking abilities of the child, making her completely dependent on her caregivers, preventive intervention is usually advised[12]. Regularly implemented standing and walking is also recommended[12,34] in order to prevent osteopenia and osteoporosis due to low bone mineral density, typical of individuals with RS[25,26,27]. When imbalance of muscle action around the ankles starts to cause muscle shortening or even joint deformation, a daily prevention program should be introduced. Such a program should include preventive splinting, daily standing in a standing frame, on a wedge so that the toes are higher then the heels (see Fig. 5).

Daily manual stretching exercises can be beneficial in this situation and the use of active splinting is recommended (see Fig. 6).

If all those preventive measures fail, serial casting can be suggested. In severe cases, the orthopedic surgeon may suggest Botox (Botulinum toxin) shots or even surgery[53]. It is mandatory that such intervention programs are followed by intensive physical therapy[12].

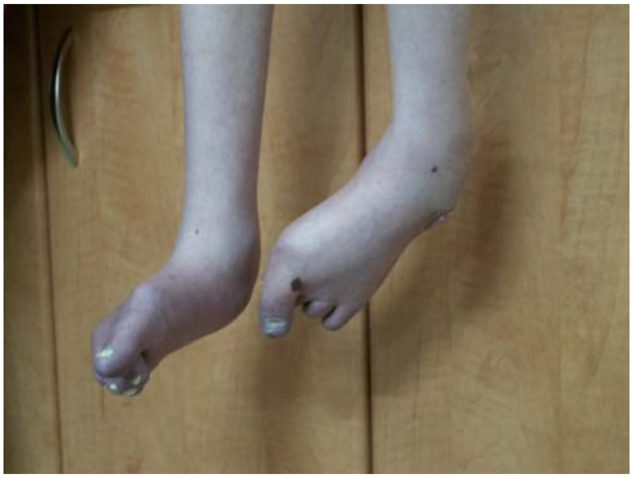

**FIGURE 4.** Feet deformities.

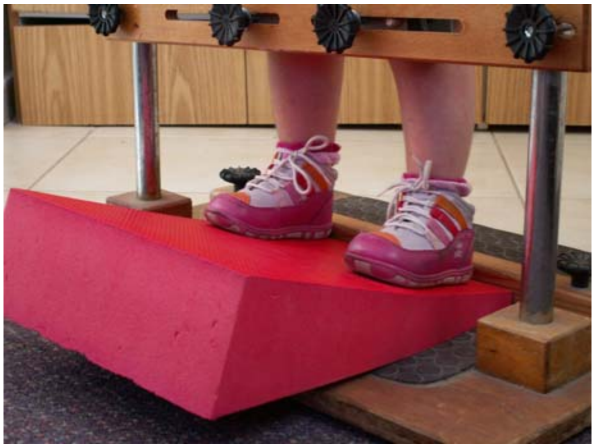

**FIGURE 5.** Stretching the gastrocnemius muscle while standing.

In the hip joint, the adductor or flexor muscles are occasionally pulled excessively tight. This can impede walking or even prevent the child from resting the hip in an extended position. Such a process left untreated over time may end up as dislocation of the hip. The tightness in these muscles should be noted, and the physical therapist should advise the parents to consult with an orthopedic surgeon in order to follow the development of such tension over time and make sure there is no development of dislocation. In the case of such muscle tightness, the physical therapist should institute a daily positioning program carried out by the educational team and/or the parents, including prone lying and positioning that will maintain hip abduction (seating on a roll). Hippotherapy, beside its other advantages, is beneficial in maintaining hip abduction[54].

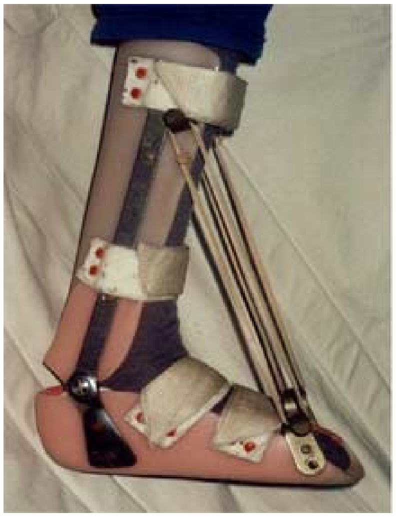

**FIGURE 6.** An active stretching splint.

## Spinal Deformities

The most common deformities seen in RS involve the spine (see Fig. 7). Such a predicament can be found in 80–85% of adults with RS[3,33]. Due to its frequency, all girls with RS should be checked for spinal curvature during their yearly physical examination. Another problem involving scoliosis is its rapid progression, averaging on a 14-degree annual rate[3,33]. The prognosis of scoliosis development in RS is worse when the scoliosis appears before age five, when severe hypotonia exists from childhood, when there is an inability to walk, or when walking was gained, but lost at an early age[3]. When such circumstances appear, follow-up of the spinal curvature should be intensified, the frequency of the child's visitations to the orthopedic surgeon increased, and therapeutic intervention applied accordingly[4].

It has been reported that 25% of mobile females with RS and 61% of nonmobile females presenting scoliosis will eventually require surgical intervention[33].

Scoliosis and kyphosis often begin with muscle tone problems, which are then enhanced by the child's inability to process her body scheme correctly (orientation in space). Active exercise and passive range of movement routines are helpful. Maintaining spinal alignment is important and can be facilitated by activities designed to provide proprioceptive (alignment of the body), kinesthetic (sensations received from joints and muscles), and tactile (touch) input thereby enhancing the individual's awareness[43].

The common intervention approach for treating scoliosis is usually a combination of the following techniques:

- Intensive physical and hydrotherapy treatments were reported to yield maximal benefits, according to the results of a questionnaire completed by 107 parents and caregivers in the U.S.[33].
- Aggressive treatment starting as soon as the first spinal asymmetry is noticed was suggested by McClure et al.[55] who found a close correlation between the primary asymmetry of the trunk and the later developing scoliosis, according to 262 responses to a questionnaire completed by families.
- Intensive walking or standing, if the individual is nonmobile, was suggested by Weeks[34], for at least half an hour a day. This element of the program is also supported by the findings by other authors regarding a relation between walking and reduced percentage of spinal operations[33].

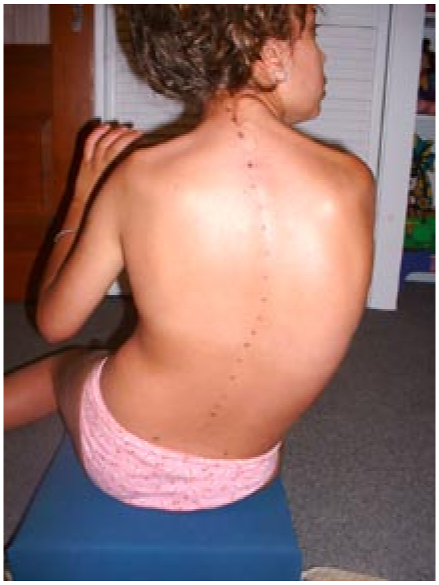

**FIGURE 7.** A child with RS and scoliosis.

- “Overcorrection” treatment, suggested by Hanks[13,32], is applied in order to readjust the skewed midline perception of the females manifesting scoliosis dominated by their skewed sensory system. There are reports of four to five cases where the progression of scoliosis was arrested using this method[48].
- Active antiscoliosis regime — This intervention has managed to reverse the scoliosis in a child with RS from 30 to 20 degrees. Within a year from intervention cessation (due to transfer of the child to a different educational facility), the spinal curvature deteriorated to 40° and the child was administered a corset[17]. This intervention is based on the following principles:
    - Visitations to an orthopedic surgeon (at least twice a year; more if the child is showing initial signs of scoliosis and is younger than 5 years)[4]
    - Commencing treatment as soon as asymmetry of the spine is detected[55]
    - Intensive implementation of the program throughout sleeping and waking hours
    - Opposing (to the natural scoliosis curve) asymmetry postures (see pictures)
    - Walking and/or standing at least 2 h/day[34]
    - Maintaining spinal mobility through passive manual manipulation
    - Parental and staff guidance[17]

This intervention regime addresses the sensory and musculature deviations associated with scoliosis in RS and tries to balance the child’s asymmetrical activity through use of various positioning equipment (see Figs. 8–12).
- Corsets and temporary casting, when used, were mostly reported as unsuccessful in slowing the progression of scoliosis in people with neuromuscular scoliosis in general and in females with RS in particular[33,56]. Nevertheless, corsets may be used on occasion in order to erect the child when functional performance is called for.
- When everything else fails, a surgical solution may be presented. It should be noted that the success of the surgical intervention depends on the spine’s flexibility and also that the postsurgical spinal curves can change up to 40–60% of the presurgical curves[33], meaning that regardless of the scoliosis management approach chosen, it is the responsibility of the physical therapist to maintain spinal mobility and flexibility.

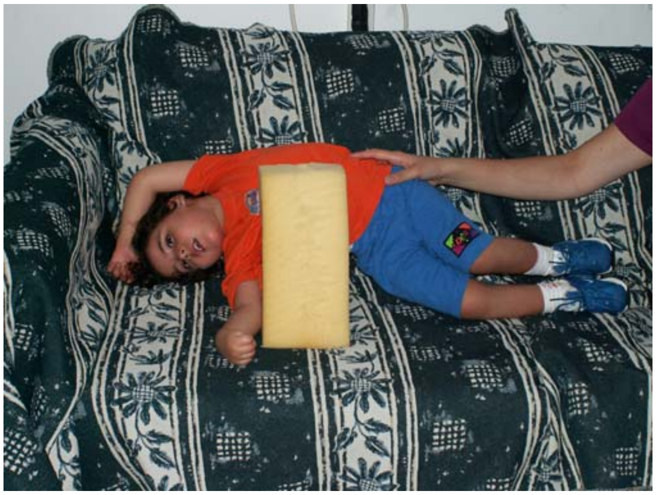

**FIGURE 8**

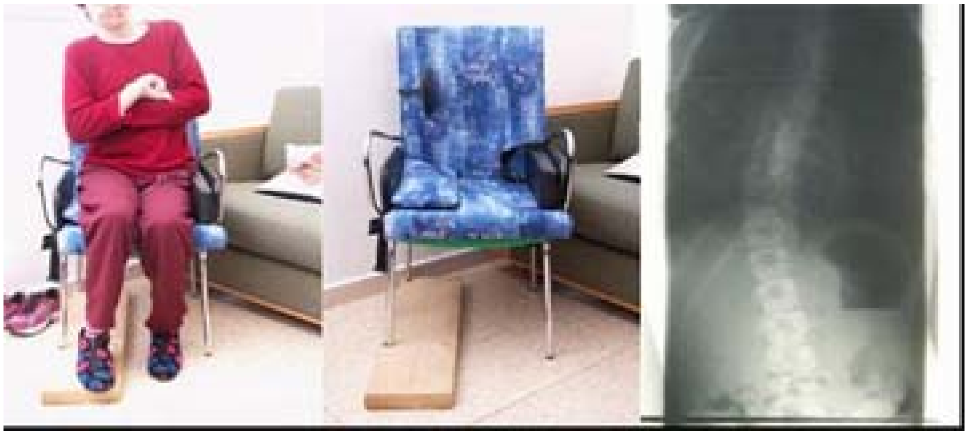

**FIGURE 9**

## IMPROVING FUNCTIONAL ABILITY AND PHYSICAL FITNESS

Ambulation or walking is a skill that requires a certain level of coordination. Of all children with RS, 50–85% achieve the ability to walk[3,57], some lose this ability later in life, some never gain it. Walking helps to prevent osteoporosis and strengthen lower extremities muscles, activates the heart and lungs, and keeps the child in shape. It is good to encourage walking in those who are able to[34], but it is sometimes impossible to teach a child to walk if the required coordination is not present[53]. Generally, it is a good idea to establish routine daily walks as a preventive measure against inactivity and a sedentary lifestyle. It

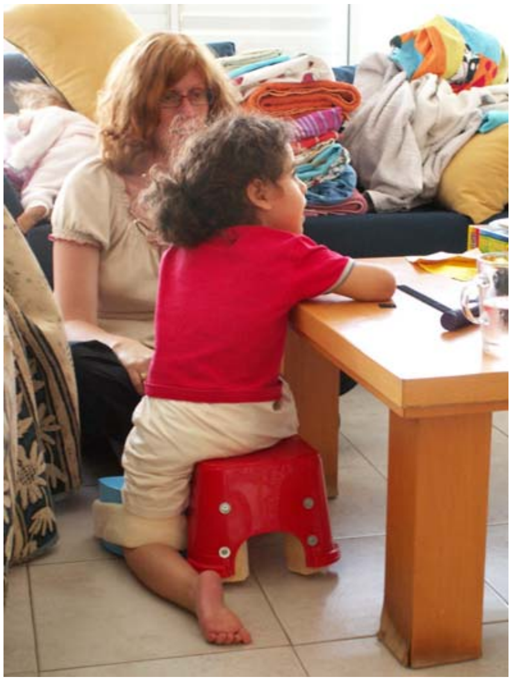

**FIGURE 10**

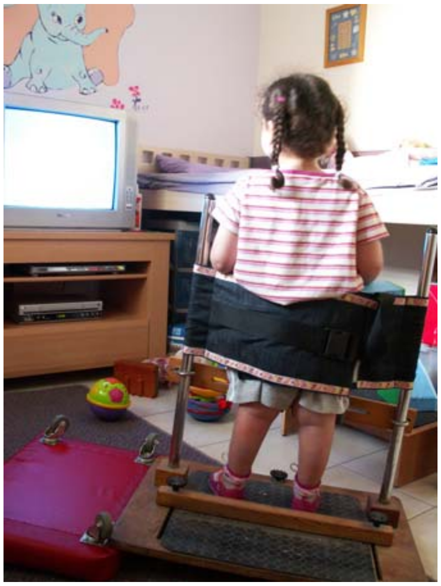

**FIGURE 11**

should be reiterated that a correlation between ambulation ability (specifically stair climbing) and milder cases of scoliosis was found[33]. In accordance with this view, a daily training program on a treadmill was designed for females with RS. The participants were four girls with RS aged 8.5–11 years (mean: 10 years) attending the educational facility Beit Issie Shapiro, Raanana, Israel. All the girls showed independent mobility and had the typical characteristics of stage III RS (see Fig. 13).

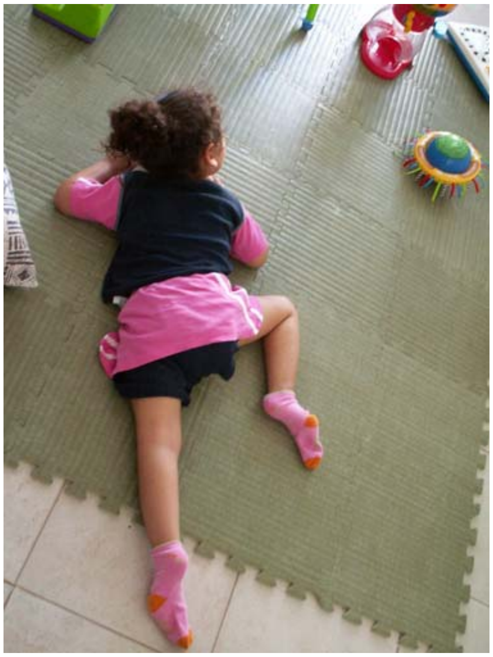

**FIGURE 12**

**FIGURES 8–12.** Different positioning equipment for management of scoliosis in RS.

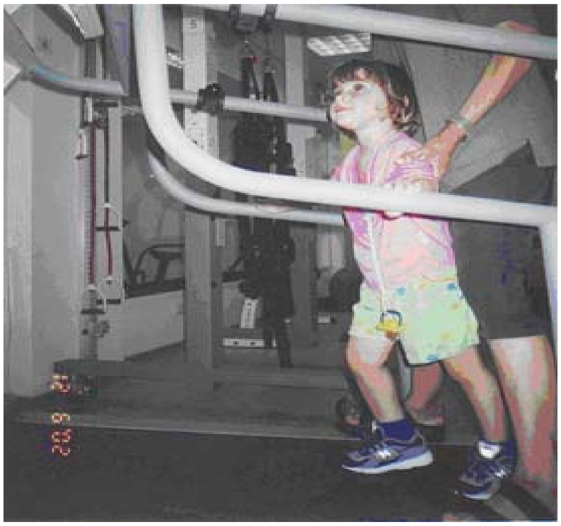

**FIGURE 13.** Treadmill training.

The intervention was executed in order to improve the physical fitness of these children and to evaluate the contribution of such an exercise program on the functional abilities of the participants. The training was conducted on a treadmill (1400 model produced by Trimline), capable of very low speeds, <0.5 km/h, with very long side rails. Special low side rails were added to the treadmill in order to fit the height of the children and Velcro straps were added to assist in the safe placement of the hands. Pulse was constantly monitored during each exercise using an A3 polar pulse transmitter belt. Since only four participants were involved and no control group was available, three tests were taken to evaluate the change derived from the intervention (test 1 was conducted 2 months before intervention was initiated, test 2 at intervention initiation, and test 3 after the completion of the intervention program). The time between tests 1 and 2 enabled detection of spontaneous changes occurring in this group without the treadmill intervention.

The intervention program was executed daily for half-hour sessions for the duration of 2 months. The results of the above-mentioned program showed that:

- Results of a two-tailed, paired, student T-test on heart rate changes between tests 1 and 2 (before intervention) showed no significant change. Heart rate reduction between tests 2 and 3 (intervention period) was found statistically significant, $p < 0.005$ (see Graphs 1 and 2). The results suggested that aerobic fitness was improved according to reduction in pulse measurements at rest and during training.

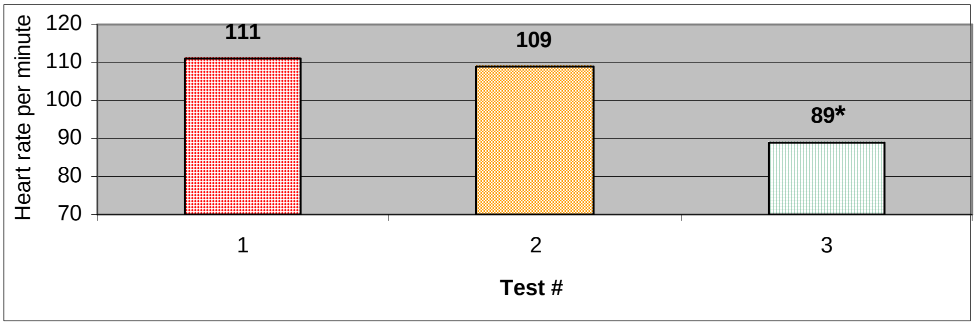

**Graph 1.** Change in resting heart rate before and after a treadmill intervention program, all participants.

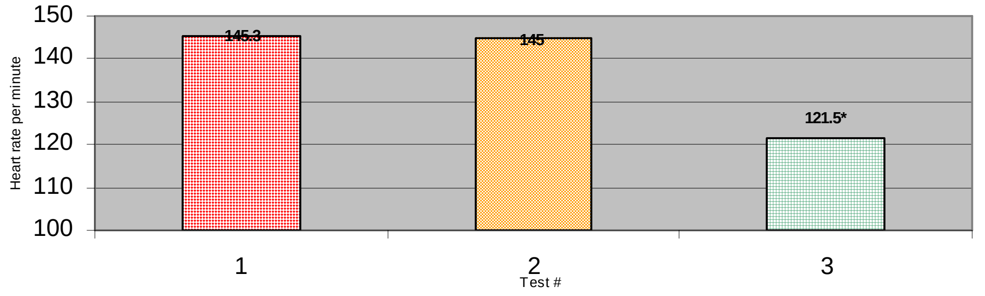

**Graph 2.** Change in exercise heart rate before and after a treadmill intervention program, all participants.

- Functional abilities were measured in the same manner: tests 1 and 2 before intervention in order to estimate spontaneous functional improvement, tests 2 and 3 at the beginning and end of the intervention period. Results show that despite lack of change in functional abilities between tests 1 and 2, functional abilities of the four participants have shown statistically significant improvement ($p < 0.0001$) between tests 2 and 3 (intervention period). Specific functional changes were observed in the ability to get up from a kneeling position to standing, walking a given distance, walking up and down stairs, and the ability to knee walk (see Graphs 3 and 4).

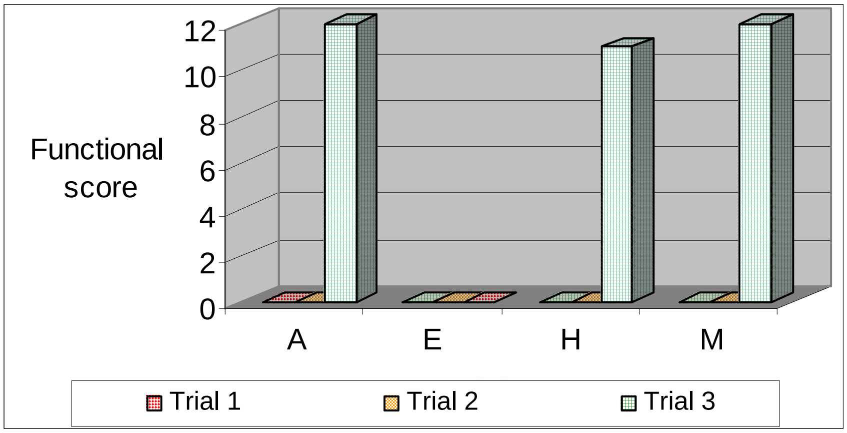

**Graph 3.** Change in stair-ascending ability tests 1–3, all participants.

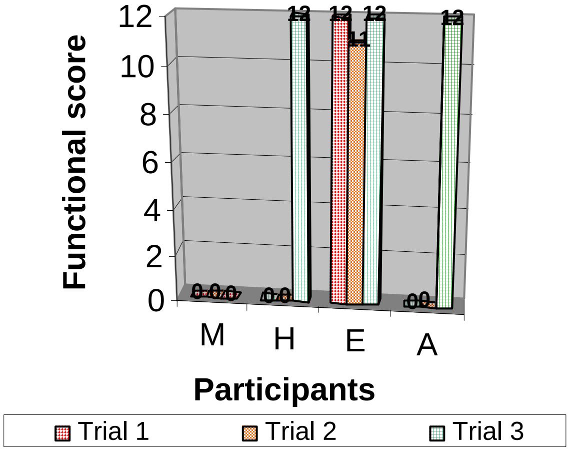

**Graph 4.** Change in knee-walking ability tests 1–3, all participants.

- When comparing the change in activity heart rate and the change in functional improvement during the treadmill intervention period (tests 2 and 3), a high (–0.76) negative and significant ($p < 0.05$) correlation was found between functional improvement and changes in heart rate. The results suggested that as heart rate levels are reduced (due to improvement in aerobic fitness), functional ability improves (see Graph 5).

The conclusions that can be drawn from the above-mentioned physical fitness program were that a daily intervention program could change the fitness level of individuals with RS and that the resulted change may improve the functional abilities of this population. The program was also found attainable by nonprofessional personnel under supervision of a qualified physical therapist[16].

It is reiterated here that information from parents disclosed that, in some cases, individuals with RS have gained walking ability at the age of 6 and even at the age of 16 years. In two different cases (studies from Sweden), two women with RS who had not walked for 15–20 years, have resumed walking ability due to an intensive program[12,15]. Due to the importance of the ambulation on health and quality of life issues, it is our belief that walking should be constantly pursued and maintained for persons with RS[4].

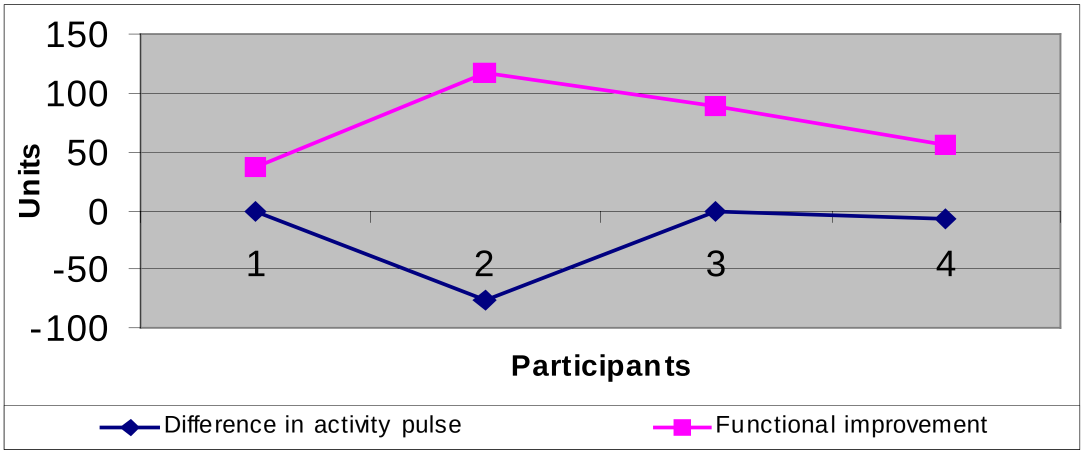

**Graph 5.** Correlation between reduction of activity heart rate to change in functional activity during treadmill intervention programs (tests 2 and 3).

### Ways of Walking

Walking stimulates the joints and muscles, and improves functional abilities and aerobic capacity[16], as well as helps the child to explore her environment. Walking with a person with RS takes patience and experience. The individual with RS will usually walk slowly and often stops or abruptly changes directions. The person with RS also has a tendency to lean on the person aiding her; therefore, it is better to stabilize the child through her shoulder while avoiding massive support[10]. Kerr and Burford have defined it as "the art of supporting the child without harming her independence"[36].

## LOSS OF TRANSITIONAL ABILITIES

Loss of transitional movements — Due to apraxia, ataxia, and sensory abnormalities, the individual with RS may find that performing what we perceive as the simplest actions, such as sitting down or lowering from kneeling to heel sitting, is impossible to them. Since loss of transitional skills is frequently seen in individuals with RS[13,32,58], daily use of all these skills should be practiced. Performing the activities of lying on her back, rolling from supine to prone and back, then moving to a hands-and-knees posture, and then standing up, can prolong or maintain the ability to independently perform these skills. Through proper instruction to the family and educational staff regarding functional abilities, for instance, standing up from sitting in a chair, can be actively practiced throughout the days, each time the child is in transit. Due to apraxia, the individual with RS might appear unable to perform transitions unless tested in a specific place and using her individual technique. In a case study, a person with RS regained transitional ability from floor to chair when the clues to her memory and motivation were found (i.e., she exercised in the same place and manner she had in the past and when it was successful under those conditions, her ability was later generalized)[12].

## OSTEOPOROSIS

Haas et al.[25] have established the fact that individuals with RS are at risk of developing osteoporosis. This group of researchers has found bone mineral density, mineral bone content, and mineral density of the spine compared between 20 young females with RS, 11 females with CP, and 25 controls with no pathology lacking. Their findings were supported by other groups as well[26]. Budden and Gunness[27]

found similar results in a study of five children with RS. They suggested that slow bone creation at a young age in individuals with RS eventually causes low bone density in this population. They hypothesized that influence of MECP2 (the gene responsible for RS) is not restricted to damage to brain tissues, and that these abnormal findings in bone development are an influence of the RS gene[27]. While the frequency of fractures has not been studied clinically in this population, it appears that there is a higher incidence in comparison to individuals without RS[58].

Osteoporosis is a multidisciplinary problem requiring proper diagnosis and organized intervention. In order to achieve appropriate therapeutic intervention, the suitable experts should be consulted (physician, as well as a dietitian and a physical therapist). The basic steps for an antiosteoporotic regime should include:

- Thorough evaluation of the child in order to sustain or eliminate a diagnosis of osteoporosis and if the osteoporosis is found present then:

- Introduction of appropriate dietetic changes
- Application of programs aimed at increased mobility, movement, and strengthening, according to the child's limitations (if the child is not mobile, an intensive daily standing exercise program should be applied – see below)
- Sunlight exposure
- Walking has been discussed at length within the content of the present article, for nonambulant individuals, standing can be used to:
    - Achieve position changes from continuous sitting
    - Prevent the appearance of pressure sores
    - Align lower extremity joints in extension
    - Maintain and increase bone calcium levels
    - Enhance circulation to internal organs
    - Provide an improved point of view on the individual's social and physical environments
    - Improve function by doing standing transfers, reducing the need to lift the client

For the prevention or at least reduction of osteoporosis, and for all the above mentioned reasons, individuals who do not walk independently should be encouraged to bear weight and practice aided walking. Children that cannot walk should be situated daily in a standing frame supported just enough to reach the client's highest postural ability[4].

## SPECIAL CONSIDERATION

### Fractures

Individuals with RS show low levels of bone density[25,26,27] and are at higher risk of receiving fractures than non-RS girls of similar age. Special care should be taken for patients who do not walk at all, are not menstruating despite their maturity, or have just come out of a cast. If the child has had several fractures, consult with a specialist about some of the newer medications, which are moderately helpful in decreasing the risk of fractures. The measurement of bone density can help with the decision regarding appropriate intervention[53]. Management of the child should include fall-prevention strategies[59], proprioceptive dynamic posture training (PDPT)[60], and proper nutritional changes[61].

Manual management for the child, especially one that has already shown pathological fractures, should always be introduced after receiving permission from her family, with careful consideration of the pros and cons of activation vs. nonactivation, after a calming explanation intended for preparing the client for the upcoming movement.

## Surgery

In cases when surgical intervention is needed (e.g., heel cord lengthening, scoliosis, Botox injection), the therapist should provide support for the family in the pre- and postsurgical management phases, as well as enhance the intensity of the physical therapy program[12]. The surgeon should be aware of the specific challenges and techniques developed for this population by relating to appropriate articles[62,63] and consulting with experienced surgeons. Follow-up instructions include recommending appropriate adaptive equipment and intervention regimes.

## Sensory Problems

Sensory problems in individuals with RS have never been investigated. Nevertheless, parents and clinicians across the world have reported different sensory abnormalities in this population[10,18]. These abnormalities should be considered when treating the individual with RS. Reduced reaction to pain was reported on numerous occasions[10] and should be taken into consideration when applying passive physical manipulations to a child with RS.

## Depth Perception

It seems that depth perception in this population is faulty, making ascending and descending stairs especially difficult. This should be taken into consideration when working on stair climbing, stair descending in particular (at such instances the therapist should stand in front of the child). With one child whom we treated, crossing over the end of a carpet was extremely difficult for her to perform and was achieved only after many hours of training and repetition.

## Tactile Hypersensitivity

Most individuals with RS present some form of hypersensitivity of the facial area, especially around the mouth[10,18]. Such sensations may put barriers on proper hygiene and eating habits, and should be dealt with with appropriate sensory desensitization. In addition to such reports of hypersensitivity around the mouth and face, the first author noticed a rejection reaction when touching the forearm of individuals with RS in several cases.

## Fear of Movement

Some children (87%) with RS display a fear of movement[30], some of them even to an extreme extent. Such fear prevents them from enjoying different activities involving movement, such as physical therapy. This fear should be handled by a sensory program applied on a regular basis. A verbal warning should be given to the child when the therapist is planning on moving her. When motion is applied, it should be done in slow movements while constantly reacting to the child’s facial expression and verbally reassuring her. It is advised to create a movement-stimulating environment around the child; raise attention to peers, introduce water games and various switches, suggest toys and sensorial stimulation, place mirrors on the walls, etc.[4].

## Concentration

In her book, Lindberg[18] mentioned that physical activity improves concentration in children with RS. This assumption should be investigated for each individual with RS under the management of the physical therapist. If found to be correct, appropriate changes in curriculum (such as individual educational intervention after physical sessions) should be made.

## RETT SYNDROME EXTREME

Although most individuals with RS show severe mobility problems, nevertheless, some have displayed extreme physical abilities, so giving in to the disabilities of RS should not be done without a struggle. This part of the article describes anecdotal cases of very active individuals with RS in the hope that such examples will inspire others involved in RS.

Motion by individuals with RS can be achieved by using a bicycle. According to parental reports, most children will have a hard time controlling the handle bars as well as the pedals, and because of this, the full function of riding a bike will be achieved with the aid of a facilitator. Although riding a bike is a normative skill, it enables the nonmobile person to experience motion and join family trips, even if "just" in a towed trailer. Mutual riding trips can be very positive experiences for both the child and her family (see Figs. 14 and 15).

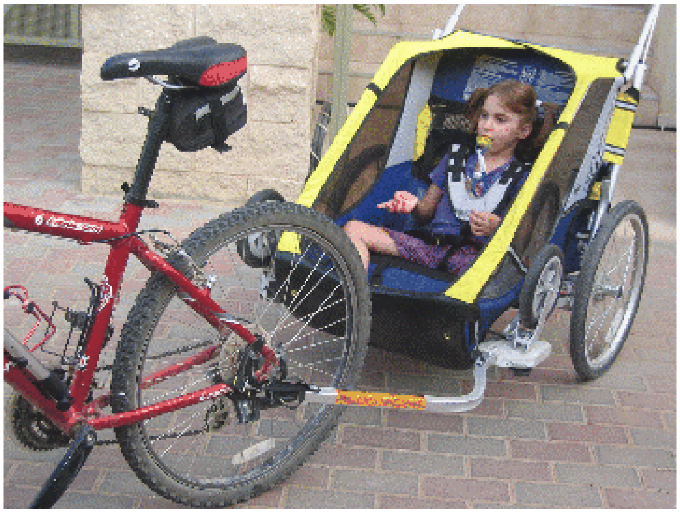

**FIGURE 14**

There are several known cases of individuals with RS mastering the art of skiing (see Fig. 16). The ability to master such a skill cannot be easily achieved by a child with RS, nonetheless, any form of activity is recommended and advised for this population

Using a trampoline is a unique tool for individuals with RS, since it facilitates extremely good muscular and postural control. An account from Australia reports of a child who practices on the trampoline on a daily basis[7]. An active daily practice routine (not necessarily on a trampoline) can improve the body scheme, fitness levels, circulation, and muscle strength of the individual and therefore is highly recommended.

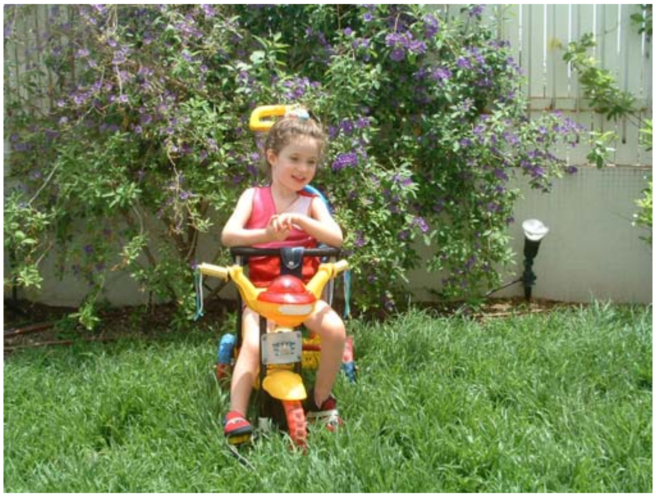

**FIGURE 15**

**FIGURES 14 and 15.** Using a bike both as a recreational and physical activity.

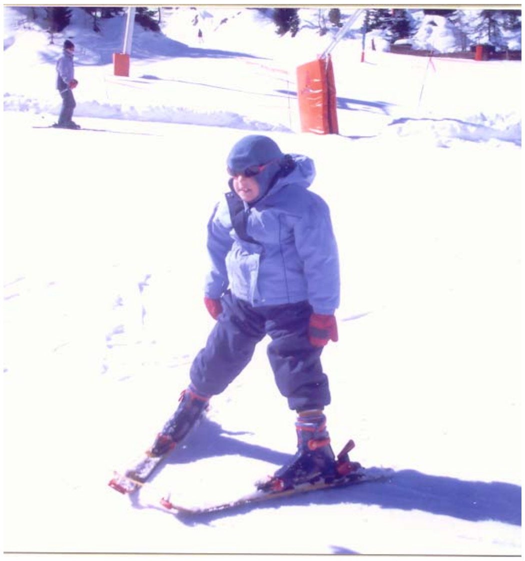

**FIGURE 16.** A child with RS skiing.

## CONCLUSIONS

The present article was written with the intention to introduce Rett syndrome (RS) to the physical therapist, familiarize them with this population, and to offer some of the authors' clinical experience in applying physical therapy intervention programs for these individuals.

Some basic guidelines are drawn in order to help initiate a physical intervention with this population. Due to the variety in phenotypic manifestation of this syndrome, individual evaluation of each client is warranted and treatment should be conducted in accordance with the findings of such an evaluation and with the available knowledge regarding RS prognosis.

The proper intervention for the individual with RS should create a net of human support in which everyone caring for her is familiarized with her needs. The authors and others who are acquainted with this disorder[12] believe that the physical therapist should be well informed regarding RS in order to apply the proper intervention program. Such a program should be conducted through well-communicated teamwork as the basic step towards answering the complex therapeutic needs of the individual with RS.

Due to the emotional sensitivity of this population, each individual with RS should be approached with care after a positive rapport has been established with the child and specific motivational factors have been established. Due to possible changes in the child's functional and medical levels, the physical therapist is advised to make routine follow-ups regarding the client's state and to adjust the intervention program in accordance with such changes.

## ACKNOWLEDGMENTS

Children and parents of individuals with RS, the Israeli Rett Center, National Evaluation Team, Chaim Sheba Medical Center, Tel Hashomer, Ramat Gan, Israel, Beit Issie Shapiro, Raanana, colleagues, and collaborator are thanked for their input and contributions.
